# Code-Free Automated Machine-Learning (AutoML) Classification of Curated MRI Images Labeled for Rare Neurological Diseases: An Image-Level Proof-of-Concept Study

**DOI:** 10.7759/cureus.117321

**Published:** 2026-10-01

**Authors:** Mark A Bachir, Neel Nawathey, Alexander Bachir, Akshay J Reddy, Jasmeen K Cheema, Rakesh Patel

**Affiliations:** 1 Internal Medicine, California Northstate University College of Medicine, Elk Grove, USA; 2 Osteopathic Medicine, Touro University California, Mare Island, USA; 3 Internal Medicine, California Health Sciences University College of Osteopathic Medicine, Fresno, USA; 4 Medicine, California University of Science and Medicine, Colton, USA; 5 Medical School, University of California, Davis, USA; 6 Internal Medicine, East Tennessee State University Quillen College of Medicine, Johnson City, USA

**Keywords:** artificial intelligence, automated machine learning, brain mri, deep learning, image classification, neuroimaging, rare neurological diseases, vertex ai

## Abstract

Rare neurological disorders present substantial diagnostic and research challenges because their low prevalence limits clinical exposure and the availability of large, well-characterized imaging datasets. Magnetic resonance imaging (MRI) plays an important role in many of these disorders because developmental, vascular, and neurodegenerative abnormalities may produce recognizable imaging phenotypes. Although deep-learning methods have demonstrated substantial potential for medical image analysis, conventional model development commonly requires programming expertise, computational infrastructure, and specialized knowledge of neural-network design. This image-level proof-of-concept study evaluated whether a commercially available code-free automated machine-learning (AutoML) platform could classify curated Joint Photographic Experts Group (JPEG) brain MRI images according to five inherited rare neurological disease labels.

The individual image, rather than the patient or MRI examination, was the unit of analysis, and the supplied labels were not independently clinically adjudicated. A publicly available Kaggle dataset initially contained 2,000 images distributed equally among Walker-Warburg syndrome (WWS), pachygyria with cerebellar hypoplasia, Moyamoya disease, pantothenate kinase-associated neurodegeneration (PKAN; labeled as Hallervorden-Spatz disease in the source dataset), and Fukuyama congenital muscular dystrophy (FCMD). Exact-file hash analysis identified 12 duplicate image contents, leaving 1,988 unique files. This procedure removed exact binary duplicates but could not identify adjacent slices, transformed or near-duplicate images, or multiple images from the same patient or examination. Google Cloud Vertex AI (Google LLC, Mountain View, CA, USA) AutoML randomly assigned 1,588 images (79.9%) to training, 200 (10.1%) to validation, and 200 (10.1%) to testing. A single-label multiclass classifier was trained using a maximum budget of eight node-hours. The model achieved an overall micro-averaged area under the precision-recall curve (AuPRC) (average precision) of 1.000, with overall precision and recall of 100% at a confidence threshold of 0.50. Each label contributed 40 test images, and all 40 in each class were correctly classified, corresponding to a class-specific correct-classification proportion of 100% (exact 95% CI, 91.2%-100.0%). Across the complete test set, 200/200 images were correctly classified, corresponding to an observed image-level accuracy of 100% (exact 95% CI, 98.2%-100.0%). These findings demonstrate workflow feasibility and separability of the supplied image labels within this curated benchmark. However, patient- and examination-level independence could not be established; the effective independent sample size may be smaller than the nominal image count, and dataset-specific technical characteristics or shortcut learning may have contributed to the observed performance. These results should not be interpreted as evidence of patient-level diagnostic accuracy, clinical robustness, or generalizability. Independent patient-level and multicenter validation using clinically representative MRI examinations is required before diagnostic relevance can be established.

## Introduction

Rare diseases collectively represent an important healthcare burden despite the low prevalence of each individual condition. Their rarity creates substantial diagnostic challenges because clinicians may encounter affected patients infrequently, while limited patient numbers and fragmented datasets complicate systematic investigation and development of computational diagnostic tools. Artificial intelligence (AI) has therefore attracted increasing interest as a means of identifying complex patterns across clinical, genetic, and imaging data relevant to rare diseases [1].

Medical imaging represents one of the most extensively investigated applications of deep learning. Deep neural networks have been applied to classification, detection, segmentation, registration, and other computer-vision tasks across multiple imaging modalities [2]. Within neuroimaging, deep-learning approaches have similarly demonstrated an ability to extract complex features from structural magnetic resonance imaging (MRI) and other imaging modalities associated with neurological disorders [3]. However, much of the neuroimaging AI literature has focused on comparatively common diseases. Rare neurological disorders remain particularly challenging because sufficiently large, representative, and independently characterized imaging datasets are difficult to obtain.

The five disorders evaluated in the present study encompass markedly different neurological and neuroradiological phenotypes. Walker-Warburg syndrome (WWS) is a severe congenital muscular dystrophy-dystroglycanopathy associated with major abnormalities of the brain, eyes, and skeletal muscle. Neuroimaging findings may include cobblestone cortical malformation, hydrocephalus, and abnormalities of the cerebellum and brainstem [4,5]. Pachygyria is a malformation of cortical development characterized by abnormally broad gyri and reduced cortical folding and may occur in association with cerebellar hypoplasia and other developmental abnormalities. Kato et al. described affected families with diffuse pachygyria and marked cerebellar hypoplasia on computed tomography and MRI, accompanied clinically by hypotonia, seizures, ataxia, and developmental delay [6]. More broadly, malformations of cortical development comprise heterogeneous genetic and imaging phenotypes for which structural MRI is central to characterization [7,8].

Moyamoya disease differs substantially from the developmental disorders represented in the dataset. It is a progressive cerebrovascular disorder characterized by stenosis or occlusion involving the terminal intracranial internal carotid arteries and their proximal branches, together with the development of abnormal collateral vascular networks [9,10]. Imaging is fundamental to diagnosis and disease characterization. MRI and magnetic resonance angiography can demonstrate parenchymal injury and vascular abnormalities, while advanced techniques may provide additional information regarding perfusion, vessel-wall characteristics, cerebrovascular reserve, and associated ischemic or hemorrhagic injury [11-13].

Pantothenate kinase-associated neurodegeneration (PKAN) is a form of neurodegeneration with brain iron accumulation associated with pathogenic variants in PANK2. A characteristic MRI feature is the “eye-of-the-tiger” appearance of the globus pallidus, consisting of central T2 hyperintensity surrounded by hypointensity related to iron accumulation [14]. The public dataset used in the present investigation labels this category as Hallervorden-Spatz disease; that source label was retained for machine-learning reproducibility, while PKAN is used throughout the manuscript when referring to the contemporary clinical entity.

Fukuyama congenital muscular dystrophy (FCMD) is an autosomal recessive dystroglycanopathy caused by pathogenic variants in fukutin (FKTN). It is characterized by muscular dystrophy in association with abnormalities of brain development, including cobblestone lissencephaly and cerebral and cerebellar dysplasia [15]. Neuroradiological investigations have additionally described cortical thickening and irregularity, polymicrogyria, pachygyria, and other developmental abnormalities [16-18].

The substantially different anatomical and pathological processes represented by these five categories make the dataset useful for proof-of-concept multiclass image classification. At the same time, the rarity of these disorders illustrates an important practical limitation of conventional deep-learning research: clinical investigators may possess relevant medical expertise without the programming experience or computational resources necessary to design and train custom neural networks.

Automated machine-learning (AutoML) offers a potential means of lowering this technical barrier. Faes et al. demonstrated that healthcare professionals without coding experience could use automated deep-learning software to develop medical image classifiers [19]. Korot et al. subsequently compared multiple code-free platforms across medical imaging datasets and demonstrated that several commercially available systems could achieve strong classification performance [20]. Such platforms abstract components of neural-network architecture selection and optimization from the end user, allowing investigators to focus on data preparation, model evaluation, and interpretation.

The present study therefore aimed to develop and internally evaluate a code-free AutoML model, defined here as a model developed through the Google Cloud Vertex AI (Google LLC, Mountain View, CA, USA) graphical interface without investigator-written machine-learning code, manual neural-network architecture design, or manual hyperparameter optimization, for multiclass classification of MRI images representing five rare neurological disorders. In addition to model development, the study incorporated explicit file-level duplicate detection because data leakage can substantially inflate apparent performance in small public imaging datasets. We hypothesized that Google Cloud Vertex AI AutoML could distinguish the five labeled image categories without investigator programming or manual neural-network architecture design. The resulting performance was interpreted as an assessment of technical feasibility and dataset separability rather than clinical diagnostic accuracy.

## Materials and methods

Study design

This proof-of-concept study evaluated a code-free deep-learning workflow for multiclass classification of rare neurological disease MRI images using Google Cloud Vertex AI AutoML. The work was conducted through California Northstate University College of Medicine, Elk Grove, California, United States. The workflow consisted of acquisition of a publicly available curated image dataset, assessment of dataset integrity, removal of exact duplicate image content, preparation for cloud-based model development, importation into Google Cloud, automated model training, internal evaluation, deployment, and demonstration of endpoint-based prediction.

No custom neural-network architecture or machine-learning training code was developed by the investigators. Reporting was structured with consideration of recommendations for transparent medical-imaging artificial intelligence research, including the Checklist for Artificial Intelligence in Medical Imaging (CLAIM) and Transparent Reporting of a multivariable prediction model for Individual Prognosis Or Diagnosis (TRIPOD)+AI [21,22].

The unit of analysis was an individual curated Joint Photographic Experts Group (JPEG) image rather than a patient or complete MRI examination. The study therefore evaluated image-level classification of labels supplied by the public dataset and did not evaluate independently confirmed patient diagnoses. No patient-level diagnostic performance was assessed.

Dataset

The Rare Neurological Diseases MRI - Curated Edition dataset developed by Muhammad Ahsan was obtained from Kaggle [23]. The curated dataset was derived from the Benchmark Diagnostic MRI and Medical Imaging Dataset published by Ajmain, Khatun, and Khushbu [24].

The curated dataset initially contained 2,000 JPEG images distributed equally among five disease categories, with 400 images per class: WWS, pachygyria with cerebellar hypoplasia, Moyamoya disease, Hallervorden-Spatz disease/PKAN, and Fukuyama muscular dystrophy.

The source dataset was distributed in predefined training, validation, and test folders containing 1,400, 300, and 300 images, respectively. Within each partition, images were organized into disease-specific folders.

Disease labels were inherited directly from the publicly available curated dataset and were retained during machine-learning import without investigator modification. The hallervorden_spatz_disease class label was retained computationally for reproducibility, although PKAN is used when discussing the contemporary clinical disorder. The investigators did not independently verify these labels using original clinical records, genetic testing, pathology, or expert neuroradiology adjudication because those source-level materials were not available with the public dataset. Accordingly, the model was trained to reproduce the supplied dataset labels rather than independently confirm clinical diagnoses. Patient and examination identifiers were also unavailable; therefore, independence of images at the patient or examination level could not be verified.

The curated dataset was derived from the Benchmark Diagnostic MRI and Medical Imaging Dataset published by Ajmain et al. [24]. According to the original Mendeley Data record, the parent dataset contains 34,192 medical images spanning 40 disease classes and was assembled from images sourced from multiple hospitals. The five disease categories relevant to the present study comprised 4,248 images in the parent dataset: 808 WWS images, 592 pachygyria with cerebellar hypoplasia images, 864 Moyamoya disease with intraventricular hemorrhage images, 1,064 Hallervorden-Spatz disease/PKAN images, and 920 Fukuyama muscular dystrophy images [24]. The Rare Neurological Diseases MRI - Curated Edition subsequently selected 400 images from each of these five categories, yielding 2,000 images in total. According to the curated dataset documentation, images were ranked using automated quality assessment based on contrast, sharpness, and brightness, and the 400 highest-quality images within each disease category were retained [23].

The publicly available source documentation does not report the number of unique patients or imaging examinations represented within either the parent or curated dataset, nor does it provide patient-level linkage between images. Although the parent dataset is described as having been sourced from multiple hospitals, the specific contributing hospitals, method of case ascertainment, number of patients per disease, number of images or slices per patient, scanner manufacturer, magnetic field strength, MRI sequence type, and detailed acquisition protocols are not provided in the public documentation. Accordingly, patient-level and examination-level independence cannot be established from the available metadata. These provenance limitations are particularly important when interpreting the high internal classification performance observed in the present study.

Consequently, independence of images at the patient and examination levels could not be verified. This represents a major methodological limitation because images originating from the same patient or examination could have been distributed across the randomly generated training, validation, and test partitions, potentially resulting in information leakage and overly optimistic estimates of model performance.

Dataset integrity assessment and duplicate removal

Before model development, the dataset was assessed for exact duplicate image content. MD5 hashes were generated for all 2,000 JPEG files and compared across the source training, validation, and test directories. This procedure identified files with identical content but could not identify visually similar images, altered copies, adjacent slices, or different images originating from the same patient. MD5 hashing identifies files with identical binary content only. It does not identify adjacent MRI slices, transformed or recompressed versions of the same image, perceptually similar or near-duplicate images, or different images originating from the same patient or examination. No perceptual-hash or image-similarity analysis was performed in the present study. Consequently, exact-file deduplication established only exact-file uniqueness and did not establish patient- or examination-level independence.

Hash analysis identified 12 duplicate image contents, leaving 1,988 unique images. The removed duplicate instances were distributed across all five disease categories: three Fukuyama muscular dystrophy images, two Hallervorden-Spatz disease/PKAN images, three Moyamoya disease images, two pachygyria with cerebellar hypoplasia images, and two WWS images. With respect to the source dataset partitions, 10 duplicate instances were removed from the original training partition and two from the validation partition; none were removed from the original test partition. When identical content occurred across multiple source partitions, retention priority was assigned in the order test, validation, and training. Several identical files occurred across the source dataset's predefined machine-learning partitions and therefore represented a potential source of data leakage if left unresolved.

A deduplicated import manifest was constructed so that only one instance of each exact image content was retained. When identical image content appeared in more than one of the source dataset partitions, only one copy was retained. A predefined retention order of test, validation, and then training was used solely as a deterministic rule for duplicate resolution. Because all deduplicated images were subsequently pooled and randomly repartitioned by Vertex AI, this retention hierarchy did not influence the final training, validation, or test assignments. After duplicate resolution, 1,390 images remained from the original training directories, 298 from the validation directories, and 300 from the test directories. Accordingly, the subsequent random partition established separation at the exact-file level only. Because patient and examination identifiers were unavailable, correlated but nonidentical images could have appeared across training, validation, and test partitions.

Class balance was preserved after deduplication. The resulting dataset contained 397 Fukuyama muscular dystrophy images, 398 Hallervorden-Spatz disease/PKAN images, 397 Moyamoya disease images, 398 pachygyria with cerebellar hypoplasia images, and 398 WWS images.

The original source partition assignments were not retained for final model development because exact duplicate image content had been identified across those partitions, indicating that the predefined split could not be assumed to be fully independent. After duplicate removal, the 1,988 unique images were pooled, and Vertex AI generated a new random approximately 80/10/10 training, validation, and test split as part of the AutoML workflow. This repartitioning was intended to provide a clean file-level split after deduplication, not to establish patient-level independence. Because patient and examination identifiers were unavailable, correlated images from the same patient or examination could still have been distributed across development and test sets. Following deduplication, all 1,988 unique images were imported into Vertex AI, which performed a new random approximately 80/10/10 training, validation, and test split. A user-specified random seed or reproducible partition identifier was not available through the AutoML workflow used in this study and was therefore not recorded. Consequently, the exact image-level partition cannot be independently reconstructed from the available study records, representing an additional limitation of reproducibility.

Image preprocessing

No additional investigator-directed segmentation, normalization, cropping, image enhancement, or data augmentation was performed before import into Vertex AI. The curated JPEG images supplied by the public dataset were used directly after exact duplicate removal. The investigators did not perform additional resizing, resampling, grayscale conversion, color conversion, normalization, cropping, or other image-standardization procedures before import. Detailed image-dimension and resolution distributions were not provided in the public dataset documentation and were not systematically extracted as part of the original workflow. Google Cloud Vertex AI performed its internal preprocessing automatically as part of the proprietary AutoML pipeline; however, the exact resizing or normalization operations applied internally were not exposed to the investigators. Consequently, class-specific differences in image dimensions, resolution, color format, or other acquisition-related characteristics could have provided unintended cues to the classifier and represent a limitation of the study.

According to the dataset documentation, the distributed images had previously undergone automated quality-based curation using measures including contrast, sharpness, and brightness [23]. The documentation describes this as the general curation approach for the dataset; however, sufficiently detailed class-specific preprocessing parameters, thresholds, or intermediate quality scores were not available to independently verify that the procedure was implemented identically across all five disease categories. Consequently, class-dependent differences introduced during source-dataset curation cannot be excluded as potential shortcut features. The present investigation therefore evaluated an already processed public image dataset rather than raw Digital Imaging and Communications in Medicine (DICOM) examinations acquired prospectively in routine clinical practice.

Accordingly, model performance in the present study represents performance on this specific curated image dataset and should not be extrapolated directly to raw clinical MRI examinations.

Google Cloud storage and dataset import

The deduplicated dataset was uploaded to Google Cloud Storage. A CSV import manifest was generated containing the Google Cloud Storage URI and corresponding disease label for each image.

All 1,988 unique labeled images were imported into Google Cloud Vertex AI as a single-label image-classification dataset. Vertex AI subsequently performed a new random approximately 80/10/10 partition for model development.

AutoML model development

A multiclass image-classification model was developed using Google Cloud Vertex AI AutoML in the us-central1 region through the Google Cloud Console graphical interface.

Incremental training was disabled because this was the first model version developed for the dataset. Model development was performed using Google Cloud Vertex AI AutoML Image Classification through the Google Cloud Console graphical interface in the us-central1 region. The task was configured as single-label multiclass image classification. The user-selectable settings recorded for this training run included incremental training disabled and a maximum training budget of eight node-hours, which was the minimum available budget for the selected workflow. No custom neural-network architecture, transfer-learning implementation, optimizer, learning rate, or investigator-directed hyperparameter settings were specified. Vertex AI automatically performed the underlying architecture selection, preprocessing, training, and optimization procedures. The final dataset was automatically partitioned into 1,588 training, 200 validation, and 200 test images, and training required one hour and 53 minutes. Because Vertex AI AutoML is a managed proprietary service, a specific underlying neural-network architecture or model-version identifier was not exposed to the investigators or recorded in the study materials. Because Google Cloud Vertex AI AutoML is a managed proprietary platform, the final neural-network architecture and complete internal hyperparameter-search strategy were not exposed to the investigators. To improve reproducibility, all platform configuration details available from the study record are reported, including use of Vertex AI AutoML Image Classification through the Google Cloud Console, the us-central1 region, single-label multiclass classification, incremental training disabled, an eight-node-hour maximum training budget, automated approximately 80/10/10 data partitioning, and the resulting training duration of one hour and 53 minutes. Platform-controlled architecture selection, preprocessing, optimization, and other internal training parameters were not available for investigator specification or export.

No custom neural-network architecture, transfer-learning implementation, optimizer selection, learning-rate selection, or manual hyperparameter tuning was performed by the investigators.

Training was initiated using a maximum computational budget of eight node-hours, corresponding to the minimum budget available for the selected AutoML workflow. The general training procedure followed Google Cloud's documented Vertex AI image-classification workflow [25].

Vertex AI randomly assigned the 1,988 unique images into 1,588 training images (79.9%), 200 validation images (10.1%), and 200 test images (10.1%). The internally held-out test set contained 40 images from each of the five disease categories: Fukuyama muscular dystrophy, Hallervorden-Spatz disease/PKAN, Moyamoya disease, pachygyria with cerebellar hypoplasia, and WWS. Model training required one hour and 53 minutes.

Model evaluation

Following completion of training, model performance was evaluated using the internally held-out Vertex AI test partition containing 200 images, with 40 images from each of the five supplied disease-label categories. Vertex AI reported overall average precision as the overall micro-averaged area under the precision-recall curve (AuPRC), with class-specific average precision reported separately for individual labels. Precision and recall were additionally evaluated at a confidence threshold of 0.50. The 0.50 threshold was used as the reported operating point and was not used to exclude images from the test cohort; all 200 images remained part of the evaluation. Because complete per-image prediction scores were not independently exported, confidence intervals for average precision itself were not calculated. The row-normalized confusion matrix showed 100% diagonal classification for all five classes. Because each class contained 40 test images, the corresponding absolute counts were 40 correct classifications and 0 off-diagonal classifications per class, yielding 200/200 correct image-level classifications overall. Exact two-sided 95% binomial confidence intervals were calculated for these observed correct-classification proportions.

Vertex AI automatically generated classification-performance metrics, including overall average precision, precision, recall, precision-recall analysis, confidence-threshold analysis, class-specific average precision, and a row-normalized confusion matrix.

Overall model performance was summarized using the metrics reported by Vertex AI. Class-specific performance was additionally examined to determine whether classification performance differed among the five disease categories.

Raw per-image prediction outputs from the complete test cohort were not independently exported. However, because the internally held-out test set contained 40 images from each of the five disease categories and the row-normalized confusion matrix showed 100% diagonal classification with no off-diagonal assignments, the corresponding confusion-matrix counts could be determined directly. Each disease category had 40 correct classifications and 0 misclassifications, yielding 200/200 correct classifications overall. The observed class-specific correct-classification proportion was therefore 100% (40/40; exact 95% binomial CI, 91.2%-100.0%) for each disease class, while overall accuracy was 100% (200/200; exact 95% binomial CI, 98.2%-100.0%). These confidence intervals quantify sampling uncertainty around the observed classification proportions; confidence intervals for average precision were not calculated because the complete per-image prediction scores were not retained.

Endpoint deployment and prediction

Following internal evaluation, the trained AutoML model was deployed to an online prediction endpoint within Google Cloud Vertex AI. Representative MRI images were submitted through the integrated prediction interface, and Vertex AI returned a predicted disease class together with an associated confidence score for each image.

One representative image from each disease category was used to demonstrate endpoint functionality. These examples were intended only to demonstrate deployed inference and were not treated as an independent validation cohort.

Statistical analysis

The study was designed as a descriptive proof-of-concept investigation. Classification metrics were generated automatically by Google Cloud Vertex AI AutoML. No inferential hypothesis testing was performed. The principal dataset characteristics, training parameters, and internal model-performance measures are summarized in Table 1.

**Table 1:** Dataset characteristics, AutoML training parameters, and internal model performance. Summary of the original and deduplicated image dataset, the final Google Cloud Vertex AI (Google LLC, Mountain View, CA, USA) training, validation, and test partitions, model-development parameters, and principal internally generated performance metrics. Performance values represent results from the internally held-out Vertex AI test set and should not be interpreted as external validation or clinical diagnostic performance. AutoML: automated machine-learning

Metric	Value
Original image files	2,000
Exact duplicate image contents identified	12
Unique images used	1,988
Training images	1,588 (79.9%)
Validation images	200 (10.1%)
Test images	200 (10.1%)
Final data partitioning	Random Vertex AI split, approximately 80/10/10
Number of classes	5
Classification task	Single-label multiclass
Maximum training budget	8 node-hours
Actual training time	1 hour 53 minutes
Overall average precision	1.000
Evaluation confidence threshold	0.50
Overall precision at threshold 0.50	100%
Overall recall at threshold 0.50	100%

## Results

Dataset integrity and model training

The initial public dataset contained 2,000 MRI image files distributed among five rare neurological disease categories. File-level hash analysis identified 12 exact duplicate image contents. Following removal of duplicate instances, 1,988 unique images remained for model development.

The five disease categories remained approximately balanced after deduplication, with 397 Fukuyama muscular dystrophy images, 398 Hallervorden-Spatz disease/PKAN images, 397 Moyamoya disease images, 398 pachygyria with cerebellar hypoplasia images, and 398 WWS images.

Following import into Google Cloud Vertex AI, the 1,988 deduplicated images were randomly assigned by the platform into 1,588 training images (79.9%), 200 validation images (10.1%), and 200 test images (10.1%). The AutoML multiclass image-classification model completed training using a maximum computational budget of eight node-hours. Actual model training required one hour and 53 minutes.

Overall classification performance

The trained classifier achieved an overall AuPRC, reported by Vertex AI as average precision, of 1.000 on the internally held-out test set. For multiclass classification, Google Cloud defines the overall AuPRC as a micro-averaged metric, aggregating predictions across all classes before calculation rather than taking an unweighted mean of class-specific values. Class-specific average precision values were obtained separately from the per-label evaluation results. At a confidence threshold of 0.50, overall precision and recall were both 100%. The 0.50 threshold was used as the operating point for reporting and was not selected through post hoc optimization of test-set performance. In Vertex AI, the confidence threshold determines whether the highest-scoring predicted label is accepted for a test image; a prediction below the threshold is treated as not meeting the assignment criterion rather than causing the image itself to be removed from the test cohort. All 200 test images remained part of the evaluation. Because recall was 100% at the 0.50 threshold, no test image was left without an accepted correct prediction at this operating point.

Precision-recall analysis similarly demonstrated an overall average precision of 1.000 on the internal test set (Figure 1).

**Figure 1:**
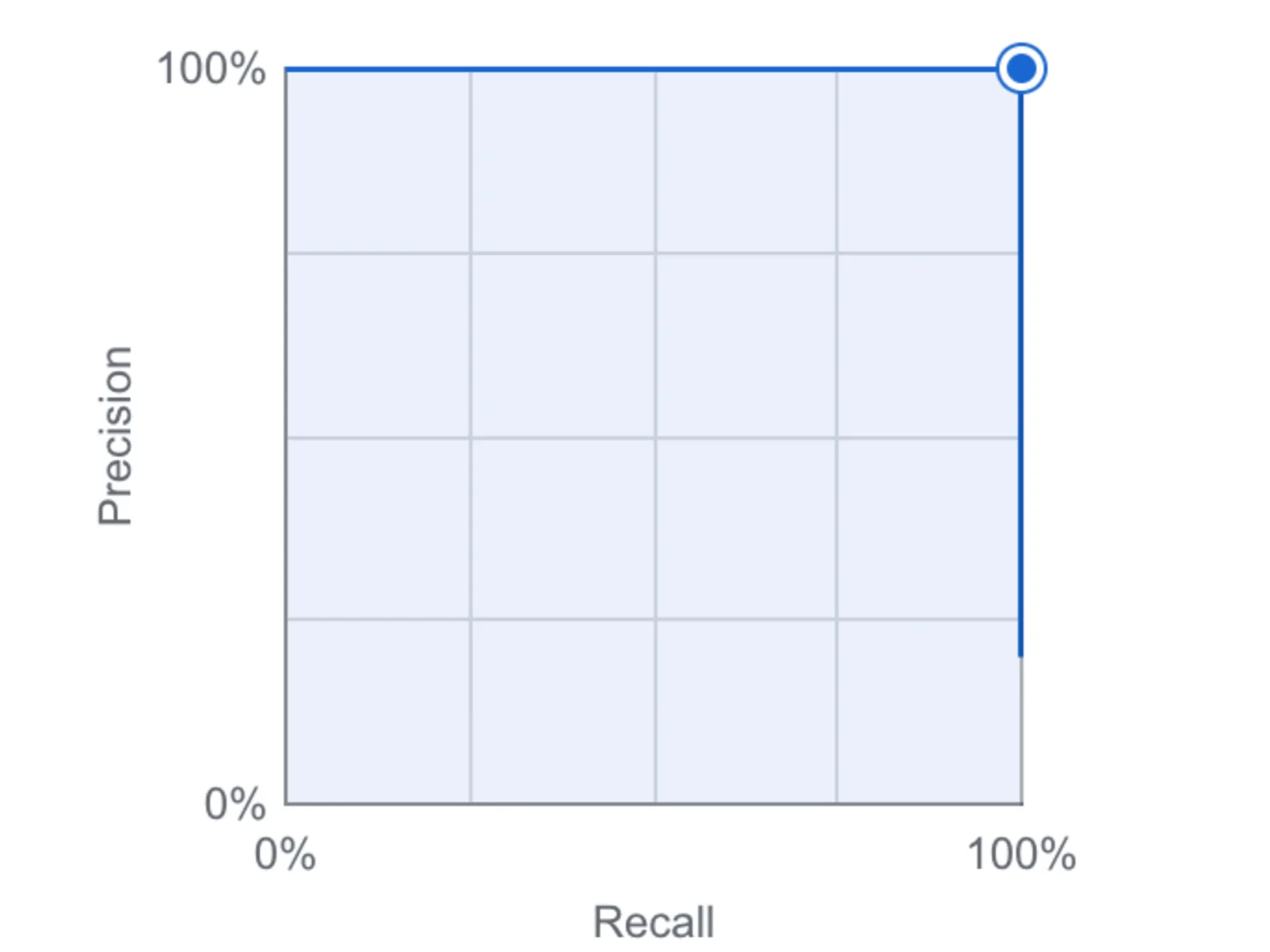
Precision-recall performance of the rare neurological disease AutoML classifier. Precision-recall curve generated by Google Cloud Vertex AI (Google LLC, Mountain View, CA, USA) for the internally held-out test set of 200 images, including 40 images per supplied disease label. Overall average precision represents the platform-reported micro-averaged area under the precision-recall curve (AuPRC). Performance represents internal image-level classification of the supplied dataset labels. AutoML: automated machine-learning

Confidence-threshold performance

Confidence-threshold analysis demonstrated high precision and recall across a broad range of operating thresholds. At the evaluated confidence threshold of 0.50, both precision and recall reached 100% (Figure 2). 

**Figure 2:**
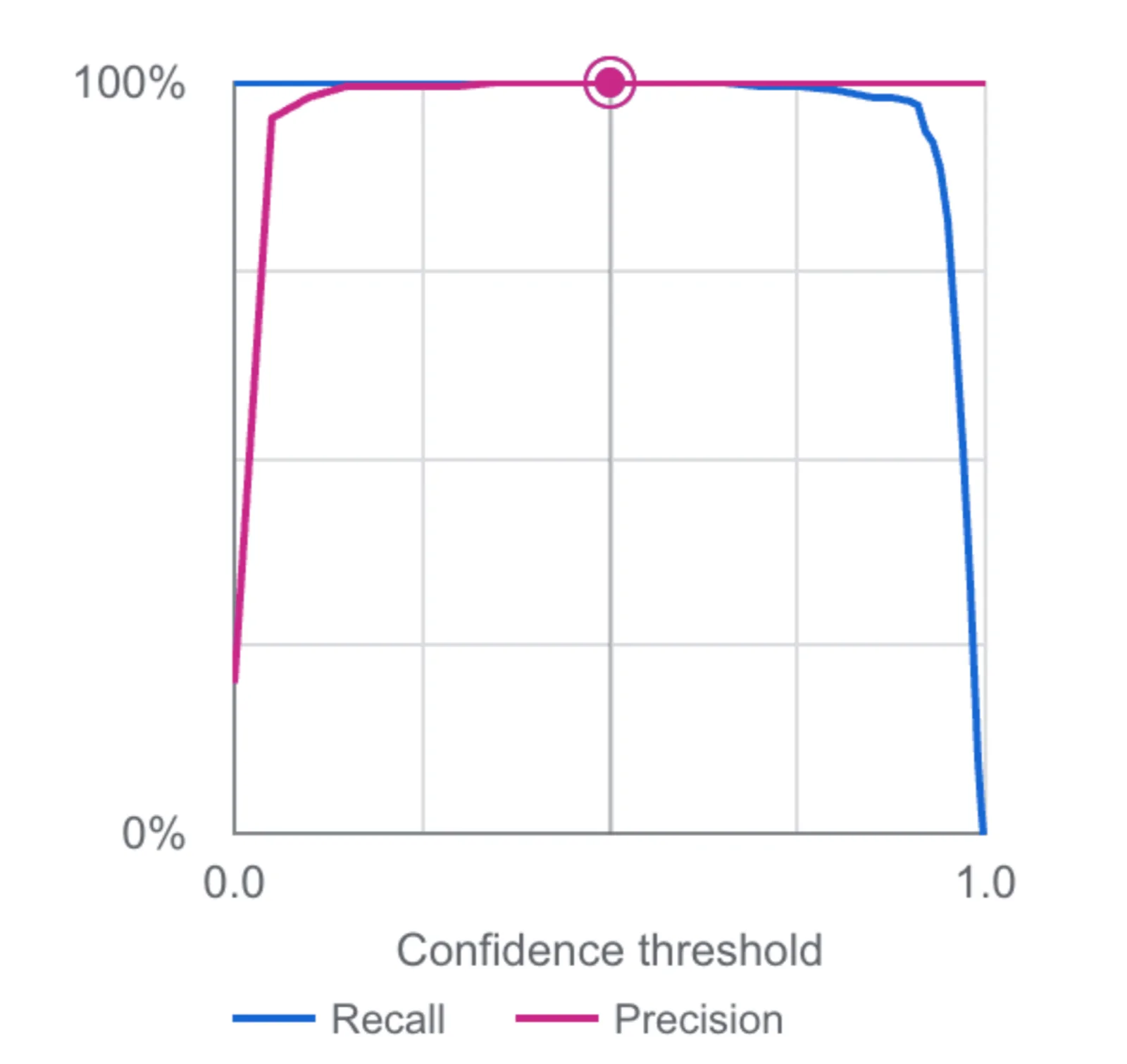
Precision and recall across prediction confidence thresholds. Precision and recall generated by Google Cloud Vertex AI (Google LLC, Mountain View, CA, USA) are plotted as functions of the prediction confidence threshold for the multiclass rare neurological disease classifier. At the evaluated threshold of 0.50, both overall precision and recall were 100%. The curves illustrate the relationship between classification confidence requirements and model performance across alternative operating thresholds. Precision and recall generated by Google Cloud Vertex AI across confidence thresholds for the internally held-out 200-image test set. A confidence threshold of 0.50 was used as the reported operating point, at which precision and recall were both 100%. All 200 images remained part of the test cohort; the threshold determined whether a prediction met the assignment criterion at a given operating point.

Class-specific performance

Class-specific average precision was 1.000 for all five disease categories, including Fukuyama muscular dystrophy, Hallervorden-Spatz disease/PKAN, Moyamoya disease, pachygyria with cerebellar hypoplasia, and WWS. No disease category demonstrated lower class-specific average precision during the internal evaluation.

Confusion matrix analysis

Row-normalized confusion-matrix analysis demonstrated 100% correct classification for each supplied disease-label category, with no observed off-diagonal assignments (Figure 3). Each category contained 40 test images; therefore, the corresponding absolute counts were 40 correct classifications and 0 misclassifications for each class, yielding 200/200 correct image-level classifications overall. Figure 3 displays row-normalized percentages, while the corresponding absolute counts are reported here in the text.

**Figure 3:**
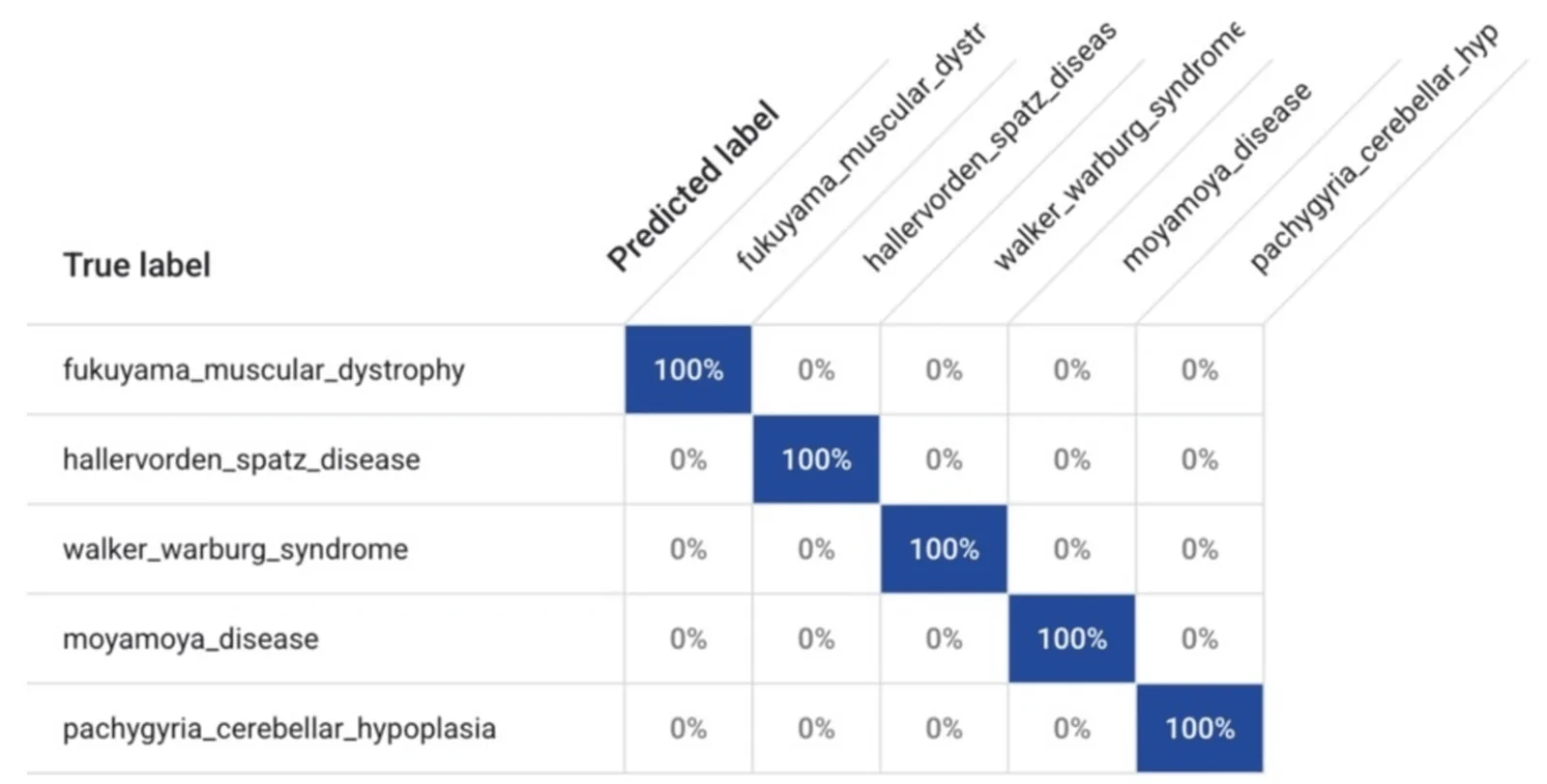
Row-normalized confusion matrix of the rare neurological disease AutoML classifier. Rows represent the supplied true disease labels and columns represent predicted labels. The matrix displays row-normalized percentages only. Each disease-label category contained 40 images in the internally held-out test set, and all 40 images in each category were correctly classified, with no observed off-diagonal assignments. The corresponding absolute counts are reported in the Results text. AutoML: automated machine-learning

Endpoint predictions

Following completion of model training and internal evaluation, the AutoML classifier was deployed to a Google Cloud Vertex AI online prediction endpoint. One representative MRI image from each of the five disease categories was submitted to the deployed endpoint solely to demonstrate online inference functionality. These images were selected qualitatively for illustrative presentation rather than through a predefined sampling procedure and were not used to calculate any model-performance metric. The training, validation, or test partition membership of these five illustrative images was not retained in the study record and therefore cannot be reliably reported retrospectively. Accordingly, these examples should be interpreted only as a demonstration of deployment functionality and not as an independent evaluation or validation cohort.

All five representative images were assigned to their corresponding disease classes. Prediction confidence scores were 0.974 for Fukuyama muscular dystrophy, 0.983 for Hallervorden-Spatz disease/PKAN, 0.917 for Moyamoya disease, 0.959 for pachygyria with cerebellar hypoplasia, and 0.956 for WWS. Confidence scores for the five illustrative endpoint examples ranged from 0.917 to 0.983 (Figure 4). These examples were selected solely to demonstrate deployment functionality and were not treated as an independent evaluation cohort or used to calculate model-performance metrics.

**Figure 4:**
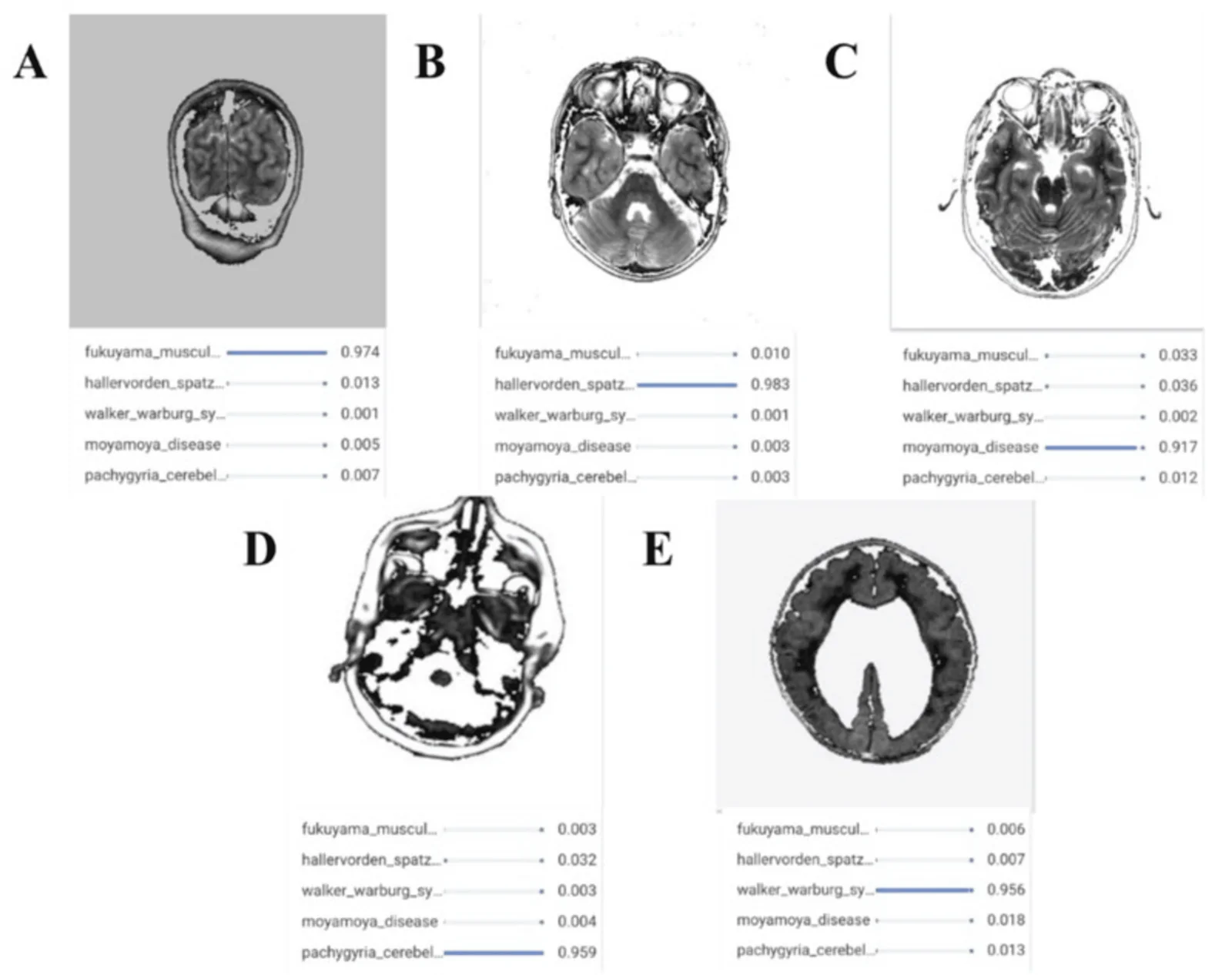
Representative predictions from the deployed Google Cloud Vertex AI endpoint. Representative online predictions generated after deployment of the trained AutoML classifier to a Google Cloud Vertex AI (Google LLC, Mountain View, CA, USA) endpoint. One MRI image from each disease category was submitted for inference. The model assigned the corresponding source class to all five examples: (A) Fukuyama muscular dystrophy, confidence score 0.974; (B) Hallervorden-Spatz disease/PKAN, confidence score 0.983; (C) Moyamoya disease, confidence score 0.917; (D) pachygyria with cerebellar hypoplasia, confidence score 0.959; and (E) Walker-Warburg syndrome, confidence score 0.956. Confidence values represent Vertex AI model-assigned prediction confidence scores. These representative examples demonstrate endpoint functionality only and were not used as an additional independent evaluation or external validation cohort. One representative image from each supplied disease-label category was submitted to demonstrate deployed inference and generation of model confidence scores. These images were selected for illustrative purposes only. Their training, validation, or test partition membership was not retained and therefore cannot be reported reliably. They were not treated as an independent validation cohort and were not used to calculate any reported performance metric. Figure 4 was created by the authors as an illustrative deployment figure using representative MRI images from the same publicly available Rare Neurological Diseases MRI - Curated Edition dataset used throughout the study and paired them with the corresponding prediction-confidence outputs generated by Google Cloud Vertex AI AutoML [23].

## Discussion

This image-level proof-of-concept study demonstrates the technical feasibility of using a commercially available code-free AutoML platform to classify curated brain MRI JPEG images according to five inherited rare neurological disease labels. Following removal of exact duplicate file content, Google Cloud Vertex AI AutoML was trained and internally evaluated using 1,988 unique image files without investigator-written machine-learning code, manual neural-network architecture construction, or manual hyperparameter optimization. The model achieved an overall micro-averaged AuPRC (average precision) of 1.000, with reported precision and recall of 100% at a confidence threshold of 0.50. Although the supplied labels were completely separated within the internally held-out image-level test set, these findings should be interpreted as evidence of workflow feasibility and benchmark label separability rather than patient-level diagnostic accuracy, clinical robustness, or generalizability.

The findings are consistent with previous work demonstrating that automated deep-learning platforms can lower technical barriers to medical image classification. Faes et al. showed that healthcare professionals without coding expertise could develop medical image classifiers using automated deep learning [19]. Korot et al. subsequently evaluated code-free platforms across multiple medical imaging datasets and demonstrated that commercially available systems could achieve strong classification performance [20]. The present investigation extends this concept to a curated multiclass dataset containing MRI images labeled for rare neurological disorders while incorporating explicit file-level dataset-integrity assessment before model development.

The five supplied disease-label categories correspond to disorders with substantially different pathological and imaging phenotypes. WWS and FCMD are dystroglycanopathies associated with developmental structural abnormalities of the brain [4,5,15-18]. Pachygyria with cerebellar hypoplasia represents a malformation of cortical development characterized by abnormal gyration and cerebellar development [6-8]. Moyamoya disease is a progressive cerebrovascular disorder associated with steno-occlusive vasculopathy and its ischemic, hemorrhagic, collateral, and hemodynamic consequences [9-13]. PKAN, in contrast, is a neurodegenerative disorder associated with basal ganglia iron accumulation and the characteristic “eye-of-the-tiger” MRI appearance [14].

These marked phenotypic differences may contribute to separation of the supplied labels. However, the present study cannot determine whether the AutoML classifier relied primarily on clinically meaningful disease-specific anatomy. The model may also have exploited systematic differences in image orientation, field of view, contrast, resolution, preprocessing, cropping, borders, background appearance, acquisition source, or other dataset-specific characteristics. A dedicated quantitative audit of these potential class-associated technical features was not performed during the original workflow. Furthermore, because the exact Vertex AI image-level partition and platform-controlled preprocessing were not retained in reproducible form, a retrospective source-dataset audit could identify potential technical differences but could not establish whether those characteristics actually drove performance in the trained model. Shortcut learning therefore cannot be excluded.

The present task also differs substantially from clinical diagnosis. Every test image belonged to one of the same five supplied label categories used during model development. The classifier was not required to distinguish these images from normal MRI examinations, common neurological conditions, other rare disorders, imaging artifacts, postoperative changes, atypical presentations, or previously unseen diagnoses. Routine clinical neuroradiology additionally incorporates complete examinations, multiple sequences and planes, vascular imaging when appropriate, prior studies, and clinical context. Accordingly, performance on this closed-set image-classification task cannot be extrapolated directly to an unselected clinical population.

A recent study by Alruwaili and Mahmood independently evaluated the same curated five-class MRI dataset using a custom multi-branch deep-learning architecture and reported near-perfect image-level classification performance [26]. The similarly high performance achieved using both a custom neural network and the code-free AutoML workflow in the present study supports the interpretation that the supplied labels in this particular benchmark are highly separable. It does not establish that either system can diagnose these disorders with comparable accuracy in independently acquired clinical MRI data. Rather, the findings further emphasize the need for patient-level external validation and careful assessment of potential dataset-specific shortcuts.

Dataset integrity and potential data leakage

A particularly important methodological finding occurred before model development. Although the source dataset contained 2,000 files and was distributed with predefined training, validation, and test folders, MD5 hash analysis identified 12 exact duplicate image contents. Several duplicates occurred across the original source partitions. If left unresolved, identical file content could have appeared in more than one development or evaluation subset and artificially inflated apparent performance.

Removal of these exact duplicate instances before model development therefore represented an important file-level quality-control measure. This finding illustrates a broader consideration in public machine-learning research: predefined dataset partitions should not automatically be assumed to be independent, and investigators remain responsible for assessing dataset integrity before model development.

However, exact-file hashing addresses only one form of possible overlap. MD5 hashing identifies files with identical binary content but cannot detect adjacent MRI slices, transformed or recompressed versions of the same image, perceptually similar or near-duplicate images, or different images originating from the same patient or examination. Patient and examination identifiers were unavailable in the curated dataset. Consequently, although exact duplicate files were removed, patient- and examination-level independence across the randomly generated training, validation, and test partitions could not be established.

This limitation is particularly important in medical imaging because correlated images originating from the same individual or examination may yield overly optimistic estimates when distributed across development and evaluation subsets. The random Vertex AI partition therefore established separation only at the exact-file level and should not be interpreted as patient-independent validation. Future investigations should use verified patient identifiers and perform partitioning at the patient level whenever possible.

Interpretation of perfect internal performance

The absence of observed classification errors in the internal test set warrants particular caution. Perfect or near-perfect performance can occur when benchmark labels are strongly separable, but in a small curated image dataset it should also prompt consideration of hidden confounding, information leakage, and shortcut learning.

Interpretation of model confidence also requires caution. Model-assigned confidence scores should not automatically be interpreted as calibrated probabilities of diagnostic correctness unless calibration has been specifically evaluated. Recent medical-imaging literature has emphasized the importance of appropriately reporting model confidence and separately evaluating calibration [27,28]. Calibration was not assessed in the present study, and the confidence scores produced by Vertex AI should therefore be interpreted only as model-generated prediction scores rather than validated probabilities of correctness.

Medical AI systems may also learn shortcut features arising from acquisition methods, preprocessing, image presentation, or other dataset-specific correlations rather than features directly related to the intended clinical task [29]. In the present dataset, potential shortcut features could include differences in image dimensions, orientation, contrast, cropping, borders, backgrounds, preprocessing patterns, or acquisition characteristics. Because these factors were not systematically audited and patient-level independence could not be established, they may have contributed to the apparently perfect image-level performance.

No saliency mapping, Grad-CAM analysis, attention visualization, or other attribution method was performed. The proprietary Vertex AI AutoML workflow did not expose the final neural-network architecture, intermediate feature maps, or trained model weights required for standard retrospective Grad-CAM implementation. Consequently, the present study cannot determine whether predictions were driven by clinically plausible neuroanatomical abnormalities or nonpathological dataset-specific characteristics.

Importantly, attribution methods would not fully resolve this uncertainty even if available. Explainability approaches such as Grad-CAM may identify image regions associated with model predictions, but they do not independently establish that the model has learned causal or clinically meaningful reasoning, and their stability and faithfulness require careful interpretation [30]. Accordingly, neither the high confidence scores nor the observed perfect internal classification establish that the classifier relied on disease-specific neuroanatomy.

Accessibility of code-free AutoML

The accessibility of the model-development workflow represents another notable aspect of this study. Conventional deep-learning research commonly requires familiarity with programming languages, neural-network frameworks, data pipelines, graphics-processing-unit infrastructure, transfer learning, optimizer selection, learning-rate schedules, hyperparameter tuning, and model deployment.

Google Cloud Vertex AI AutoML abstracts many of these processes from the end user. In the present study, investigator effort was concentrated primarily on dataset acquisition, integrity assessment, preparation, cloud import, task and training-budget selection, review of automatically generated performance metrics, deployment, and interpretation rather than manual implementation of the neural network.

Such platforms may broaden participation in exploratory biomedical AI research by allowing clinicians and investigators without extensive programming experience to evaluate the feasibility of machine-learning applications. They may also facilitate preliminary hypothesis testing before investigators commit resources to custom architecture development.

However, code-free model development does not eliminate the methodological requirements of medical AI research. An automated platform cannot determine whether a dataset is clinically representative, whether supplied labels are reliable, whether correlated images from the same patient cross partitions, whether evaluation data are truly independent, or whether excellent performance reflects clinically meaningful features rather than shortcut learning. The code-free approach also introduces a trade-off between accessibility and transparency because the underlying architecture and optimization procedures are not fully exposed to the investigator.

Limitations

Several limitations substantially restrict interpretation of these findings.

First, the dataset was relatively small, containing fewer than 2,000 unique image files across five supplied disease-label categories. Although the categories were approximately balanced, this dataset cannot represent the full clinical, demographic, technical, and phenotypic variability of these rare disorders.

The internally held-out test cohort was also small, consisting of 200 images with only 40 images per supplied disease label. This sample size supports only a preliminary image-level proof-of-concept evaluation and does not provide precise evidence of model robustness. Although 40/40 images were correctly classified within each class, the corresponding exact 95% binomial confidence interval was approximately 91.2%-100.0%. For the complete 200-image test set, the interval corresponding to 200/200 correct classifications was approximately 98.2%-100.0%. The perfect point estimates should therefore be interpreted together with these lower confidence bounds rather than as evidence of error-free performance.

Furthermore, these confidence intervals treat individual images as independent observations. Because patient and examination identifiers were unavailable, multiple images may have originated from the same individual or MRI examination. If correlated images were present, the effective independent sample size would be smaller than the nominal 200 images, and image-level confidence intervals could understate the true uncertainty. Accordingly, 100% internal image-level accuracy should not be interpreted as evidence of model robustness or patient-level diagnostic performance.

Second, the dataset was a curated derivative of a larger public medical imaging collection rather than a prospectively assembled clinical cohort. Images underwent quality-based selection before the present investigation. Such curation may create an easier and less clinically representative classification environment than routine MRI, where scanner characteristics, acquisition protocols, artifacts, image quality, disease severity, and comorbid abnormalities vary considerably. Detailed class-specific curation parameters were not available, and class-dependent preprocessing or quality-selection effects therefore cannot be excluded.

Third, patient- and examination-level independence could not be established. Exact duplicate files were removed through hash analysis, but exact hashing cannot identify adjacent slices, transformed or recompressed images, perceptually similar images, or different images originating from the same patient or examination. Correlated but nonidentical images could therefore have been distributed across the randomly generated training, validation, and test partitions, potentially introducing information leakage and inflating internal performance estimates.

Fourth, the model classified isolated JPG images rather than complete MRI examinations. Clinical MRI interpretation incorporates multiple sequences, planes, slices, vascular imaging when appropriate, prior examinations, and clinical context. A single processed image cannot reproduce the information available to a neuroradiologist reviewing a complete examination.

Fifth, the experiment used a five-class closed-set design. Every evaluated image belonged to one of the same supplied categories used during model development. The classifier was not required to recognize normal examinations, common neurological disorders, unrelated rare diseases, imaging artifacts, or previously unseen diagnostic categories.

Sixth, the disease labels were inherited directly from the public dataset and were not independently adjudicated by the investigators using original clinical records, genetic testing, pathology, or expert neuroradiology review. Accordingly, the present experiment evaluates reproduction of supplied dataset labels rather than independently confirmed clinical diagnoses.

Seventh, detailed acquisition metadata were unavailable. Scanner manufacturer, magnetic field strength, MRI sequence, institutional source, acquisition protocol, and other technical variables could not be systematically assessed as potential confounders.

Eighth, Google Cloud Vertex AI AutoML is proprietary. Although the code-free approach improves accessibility, the final neural-network architecture, complete internal preprocessing procedures, and optimization strategy were not exposed to the investigators. The absence of a retained reproducible random seed or exact partition identifier additionally prevents exact reconstruction of the final image-level split.

Ninth, no formal calibration analysis was performed. Consequently, the confidence scores generated by the deployed model should not be interpreted as calibrated probabilities of diagnostic correctness.

Tenth, no formal explainability analysis was performed. The current results therefore cannot establish whether predictions were driven by clinically meaningful anatomical findings or dataset-specific artifacts. Moreover, even if attribution maps had been available, such methods alone would not establish causal or clinically meaningful model reasoning.

Eleventh, complete raw per-image prediction outputs from the internally held-out test cohort were not independently retained for retrospective analysis. Although the known class denominators and row-normalized confusion matrix permitted reporting of absolute correct-classification counts and binomial confidence intervals, independent recalculation of score-based metrics, calibration, or alternative operating characteristics was not possible.

Finally, no independent external dataset was evaluated. Under contemporary medical-imaging AI reporting principles, the findings represent internal image-level test performance rather than external validation [21]. Generalizability to independently acquired patient-level clinical MRI data therefore remains unknown.

Future directions

Future research should evaluate code-free AutoML using substantially larger datasets with verified patient-level identifiers and independently confirmed diagnostic labels. Training, validation, and testing should be partitioned at the patient level so that images from the same individual or examination cannot appear in multiple subsets.

Independent external validation should use MRI examinations acquired at different institutions and include variation in scanner manufacturers, magnetic field strengths, pulse sequences, demographic characteristics, disease severity, and image quality. Clinically realistic comparison groups should include normal examinations, common neurological disorders, alternative malformations of cortical development, other dystroglycanopathies, other cerebrovascular diseases, other forms of neurodegeneration with brain iron accumulation, and diagnostically ambiguous cases.

Future studies should also evaluate complete MRI examinations or clinically appropriate combinations of sequences rather than isolated JPG images. Examination-level or three-dimensional approaches may more accurately reproduce real-world neuroradiology interpretation.

Prospective workflows should retain exact patient-level partition assignments, image identifiers, preprocessing information, raw prediction scores, model configuration metadata, and reproducible random seeds or partition identifiers whenever available. This would permit independent calculation of confusion-matrix counts, sensitivity, specificity, positive predictive value, negative predictive value, F1-score, receiver-operating-characteristic metrics, precision-recall metrics, calibration measures, and confidence intervals.

Calibration analysis should be incorporated to determine whether prediction confidence corresponds appropriately to observed correctness. Future studies should also prospectively audit image dimensions, aspect ratios, borders, backgrounds, preprocessing patterns, and other potential technical shortcuts across disease-label categories.

Explainability techniques such as Grad-CAM or related attribution methods may additionally help identify whether model predictions localize to clinically plausible neuroanatomical regions or obvious nonanatomical artifacts. However, such methods should be interpreted as complementary analyses rather than proof of causal or clinically meaningful model reasoning.

Finally, direct comparisons between code-free AutoML systems and manually developed neural-network architectures using identical patient-level partitions and independent external validation cohorts would help determine whether the accessibility advantages of AutoML can be achieved while maintaining adequate transparency, calibration, reproducibility, and generalizability.

## Conclusions

This image-level proof-of-concept study demonstrates the technical feasibility of using Google Cloud Vertex AI AutoML to classify inherited labels assigned to curated JPG brain MRI images representing five rare neurological disease categories without investigator-written machine-learning code or manual neural-network design. After removal of exact duplicate file content, the model achieved complete separation of the supplied labels within the internally held-out 200-image test set. However, each class contained only 40 test images; patient- and examination-level independence could not be established, and exact-file hashing could not exclude adjacent slices, transformed or near-duplicate images, or correlated images originating from the same patient or examination. In addition, model confidence was not evaluated for calibration, potential shortcut learning could not be excluded, and no independent external cohort or feature-attribution analysis was available to establish clinical generalizability or clinically meaningful model reasoning. Accordingly, the findings should be interpreted as evidence of code-free workflow feasibility and image-level label separability within this curated benchmark rather than patient-level diagnostic accuracy, clinical robustness, or generalizability. Future evaluation using larger patient-level datasets, verified diagnostic labels, clinically representative complete MRI examinations, and independent multicenter external validation is required before any diagnostic relevance can be established.

## References

[1] Nishat SM, Shahid Tanweer A, Alshamsi B (2025). Artificial intelligence: a new frontier in rare disease early diagnosis. Cureus.

[2] Litjens G, Kooi T, Bejnordi BE (2017). A survey on deep learning in medical image analysis. Med Image Anal.

[3] Zhang L, Wang M, Liu M, Zhang D (2020). A survey on deep learning for neuroimaging-based brain disorder analysis. Front Neurosci.

[4] Vajsar J, Schachter H (2006). Walker-Warburg syndrome. Orphanet J Rare Dis.

[5] Rhodes RE, Hatten HP Jr, Ellington KS (1992). Walker-Warburg syndrome. AJNR Am J Neuroradiol.

[6] Kato M, Takizawa N, Yamada S (1999). Diffuse pachygyria with cerebellar hypoplasia: a milder form of microlissencephaly or a new genetic syndrome?. Ann Neurol.

[7] Barkovich AJ, Guerrini R, Kuzniecky RI, Jackson GD, Dobyns WB (2012). A developmental and genetic classification for malformations of cortical development: update 2012. Brain.

[8] Guerrini R, Dobyns WB (2014). Malformations of cortical development: clinical features and genetic causes. Lancet Neurol.

[9] Kuroda S, Houkin K (2008). Moyamoya disease: current concepts and future perspectives. Lancet Neurol.

[10] Kuroda S, Fujimura M, Takahashi J (2022). Diagnostic criteria for Moyamoya disease—2021 revised version. Neurol Med Chir (Tokyo).

[11] Lehman VT, Cogswell PM, Rinaldo L, Brinjikji W, Huston J, Klaas JP, Lanzino G (2019). Contemporary and emerging magnetic resonance imaging methods for evaluation of Moyamoya disease. Neurosurg Focus.

[12] Shahabi M, Zareshahi N, Kiani I (2025). High-resolution vessel wall MRI in Moyamoya disease: a systematic review and meta-analysis of diagnostic and prognostic applications. Neuroradiology.

[13] Novillo-Solis C, Salvador-Orbea M, Morales-Acosta A, Leon-Rojas JE (2026). Imaging-based diagnostic approaches in Moyamoya disease: a scoping review. J Clin Med.

[14] Gregory A, Hayflick SJ (2026). Pantothenate kinase-associated neurodegeneration. GeneReviews® [Internet].

[15] Saito K (2026). Fukuyama congenital muscular dystrophy. GeneReviews® [Internet].

[16] Aida N (1998). Fukuyama congenital muscular dystrophy: a neuroradiologic review. J Magn Reson Imaging.

[17] Ishigaki K, Taniguchi-Ikeda M (2025). Fukuyama congenital muscular dystrophy: clinical features and therapeutic advances. Brain Dev.

[18] Agarwal A, Sabat S, Kanekar S (2022). Fukuyama congenital muscular dystrophy. Cureus.

[19] Faes L, Wagner SK, Fu DJ (2019). Automated deep learning design for medical image classification by health-care professionals with no coding experience: a feasibility study. Lancet Digit Health.

[20] Korot E, Guan Z, Ferraz D (2021). Code-free deep learning for multi-modality medical image classification. Nat Mach Intell.

[21] Tejani AS, Klontzas ME, Gatti AA, Mongan JT, Moy L, Park SH, Kahn CE Jr (2024). Checklist for artificial intelligence in medical imaging (CLAIM): 2024 update. Radiol Artif Intell.

[22] Collins GS, Moons KG, Dhiman P (2024). TRIPOD+AI statement: updated guidance for reporting clinical prediction models that use regression or machine learning methods. BMJ.

[23] Ahsan M (2026). Rare Neurological Diseases MRI - Curated Edition. https://www.kaggle.com/datasets/ahsanneural/rare-neurological-diseases-mri-curated-edition?.

[24] Ajmain MR, Khatun MF, Khushbu SA (2024). Benchmark Diagnostic MRI and Medical Imaging Dataset. https://data.mendeley.com/datasets/d73rs38yk6/1.

[25] (2026). Google Cloud. Train an image classification model. https://docs.cloud.google.com/vertex-ai/docs/image-data/classification/train-model?.

[26] Alruwaili M, Mahmood MA (2026). A dual-branch frequency-aware attention framework for rare neurological disease classification from brain MRI. Diagnostics (Basel).

[27] Fathi M, Vakili K, Hajibeygi R (2025). Cultivating diagnostic clarity: the importance of reporting artificial intelligence confidence levels in radiologic diagnoses. Clin Imaging.

[28] Dawood T, Chen C, Sidhu BS (2023). Uncertainty aware training to improve deep learning model calibration for classification of cardiac MR images. Med Image Anal.

[29] Ong Ly C, Unnikrishnan B, Tadic T (2024). Shortcut learning in medical AI hinders generalization: method for estimating AI model generalization without external data. NPJ Digit Med.

[30] Muhammad D, Bendechache M (2024). Unveiling the black box: a systematic review of explainable artificial intelligence in medical image analysis. Comput Struct Biotechnol J.

